# Case reports of two pedigrees with recessive arrhythmogenic right ventricular cardiomyopathy associated with homozygous Thr335Ala variant in *DSG2*

**DOI:** 10.1186/s12881-017-0442-3

**Published:** 2017-08-17

**Authors:** Sami Qadri, Olli Anttonen, Juho Viikilä, Eija H. Seppälä, Samuel Myllykangas, Tero-Pekka Alastalo, Miia Holmström, Tiina Heliö, Juha W. Koskenvuo

**Affiliations:** 1 Heart and Lung Center HUH, University of Helsinki, Helsinki, Finland; 2 Department of Cardiology, Päijät-Häme Central Hospital, Lahti, Finland; 3 Blueprint Genetics, Helsinki, Finland; 4 Institute of Biomedicine, University of Helsinki, Helsinki, Finland; 5 Hospital for Children and Adolescents, Institute of Clinical Medicine, University of Helsinki, Helsinki, Finland; 6 Department of Clinical Physiology and Nuclear Medicine, HUS Medical Imaging Center, Helsinki University Hospital and University of Helsinki, 00290 Helsinki, Finland

**Keywords:** Arrhythmogenic right ventricular cardiomyopathy, Cardiomyopathies, Desmosomes, *DSG2*, Mutation, Case series

## Abstract

**Background:**

Arrhythmogenic right ventricular cardiomyopathy (ARVC) is an inherited cardiac disease, involving changes in ventricular myocardial tissue and leading to fatal arrhythmias. Mutations in desmosomal genes are thought to be the main cause of ARVC. However, the exact molecular genetic etiology of the disease still remains largely inconclusive, and this along with large variabilities in clinical manifestations complicate clinical diagnostics.

**Case presentation:**

We report two families (*n* = 20) in which a desmoglein-2 (*DSG2*) missense variant c.1003A > G, p.(Thr335Ala) was discovered in the index patients using next-generation sequencing panels. The presence of this variant in probands’ siblings and children was studied by Sanger sequencing. Five homozygotes and nine heterozygotes were found with the mutation. Participants were evaluated clinically where possible, and available medical records were obtained. All patients homozygous for the variant fulfilled the current diagnostic criteria for ARVC, whereas none of the heterozygous subjects had symptoms suggestive of ARVC or other cardiomyopathies.

**Conclusions:**

The homozygous *DSG2* variant c.1003A > G co-segregated with ARVC, indicating autosomal recessive inheritance and complete penetrance. More research is needed to establish a detailed understanding of the relevance of rare variants in ARVC associated genes, which is essential for informative genetic counseling and rational family member testing.

**Electronic supplementary material:**

The online version of this article (doi:10.1186/s12881-017-0442-3) contains supplementary material, which is available to authorized users.

## Background

Arrhythmogenic right ventricular cardiomyopathy (ARVC) is an inherited cardiac disorder, affecting predominantly the right and sometimes the left ventricle and is characterized by progressive fibro-fatty replacement of ventricular myocardial tissue [1–5]. Clinical manifestations include recurrent, typically exercise-related ventricular arrhythmias, syncope, heart failure and sudden cardiac death [6–8]. Initial presentation is generally at adolescence or young adulthood [3]. Diagnosis is based on the revised 2010 Task Force Criteria (TFC) by Marcus et al., establishing complex requirements for right ventricular (RV) function and structure, electrocardiographic (ECG) findings and genetic or familial background of the disease [9]. Prevalence in the general population has been estimated to be from 1:5000 to 1:1000 [10, 11].

Mutations in genes encoding cardiac desmosomal proteins are the major determinants of ARVC [12]. These include plakophilin-2 (*PKP2*), desmoplakin (*DSP*), desmoglein-2 (*DSG2*), desmocollin-2 (*DSC2*) and plakoglobin (*JUP*) genes [13]. The mode of inheritance is typically autosomal dominant with incomplete penetrance and variable expression [14]. Rare recessive forms may be seen either as sole cardiomyopathy or in conjunction with systemic cutaneous disorders with palmoplantar keratoderma and woolly hair [15–19]. Recent studies have revealed that cardiac desmosomal gene mutations also associate with dilated cardiomyopathy (DCM) phenotype [20, 21]. Mutations in the DSP gene explained 5.5% of DCM cases in a Finnish study cohort [22].

As much as 30–50% of ARVC patients carry at least one variant classified as disease causing [23, 24]. However, molecular genetic diagnostic yield is known to be highly variable, at least partly due to different variant classification practices. Variability in clinical manifestations, reduced penetrance and digenic inheritance, along with disease modifying lifestyle factors such as exercise act together in complicating the determination of conclusive inheritance patterns [25–28]. End-stage ARVC is also sometimes difficult to distinguish from DCM, with significantly overlapping morphology due to left ventricular (LV) involvement and apparent similarities in molecular genetic etiology [29]. Thus, more research is needed to reveal a group of genes associated with the ARVC phenotype, their inheritance patterns, and genotype-phenotype associations in the disease.

Here, we report two Finnish pedigrees (*n* = 20) in which a homozygous *DSG2* variant c.1003A > G, p.(Thr335Ala) co-segregated with ARVC, indicating autosomal recessive inheritance and complete penetrance. All heterozygous mutation carriers were healthy in terms of cardiac disease, even though this variant has been previously described as disease causing in a heterozygous state.

## Case presentation

The index patients of these two families fulfilled the revised diagnostic criteria for ARVC by Marcus et al. [9]. Family history was obtained and pedigrees were drawn. Our clinicians assessed the adult family members at Helsinki University Hospital or Päijät-Häme Central Hospital where possible by physical examination, resting 12-lead ECG, signal-averaged electrocardiography (SAECG), appropriate laboratory tests and transthoracic echocardiography or cardiac magnetic resonance imaging (MRI). From those already deceased, we acquired all available hospital records concerning cardiac diseases. The clinical diagnoses of the family members were also based on the 2010 TFC. All participants are of Finnish ethnicity.

### Molecular genetic studies

**Fig. 1:**
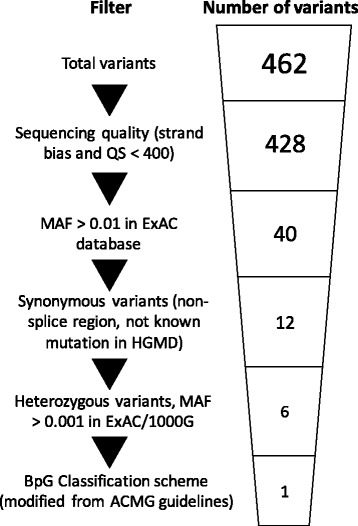
Overview of the variant filtering scheme. Filters used are listed on the left, and the number of variants on each step are depicted on the right. QS = quality score; MAF = minor allele frequency; ExAC = Exome Aggregation Consortium; HGMD = Human Gene Mutation Database; 1000G = 1000 Genomes Project; BpG = Blueprint Genetics; ACMG = American College of Medical Genetics and Genomics

Blueprint Genetics carried out genetic testing using targeted sequencing panels utilizing oligonucleotide-selective sequencing (OS-Seq) [30]. Family 1 proband’s genetic evaluation was performed using the Pan Cardiomyopathy Panel, covering 103 genes associated with pediatric and adult onset cardiomyopathies and their phenocopies. Family 2 proband was genetically evaluated using the Heart Panel, covering 133 genes associated with cardiomyopathies and hereditary arrhythmias. An additional file describes these next generation sequencing (NGS) based panels in more detail (see Additional file 1). Our variant filtering scheme is outlined in Fig. 1. We studied the presence of Thr335Ala in *DSG2* in probands’ relatives by bi-directional Sanger sequencing.

### Family screening

We found both probands to be homozygous for the missense variant c.1003A > G, p.(Thr335Ala), (rs191564916) in *DSG2*. In silico predictions for this variant are contradictory, as it is predicted to be damaging by PolyPhen [31] and Mutation Taster [32] but tolerated by SIFT [33]. There is one heterozygous carrier of the variant in the 1000 Genomes Project [34] and a total of 67 carriers in the Exome Aggregation Consortium database (ExAC, *n* = 60,275) [35]. The p.(Thr335Ala) in *DSG2* is fairly common especially among the Finnish population as six of the carriers were from Finland (n(FIN) = 3304). However, there are no homozygote individuals in the ExAC control cohorts.

**Fig. 2:**
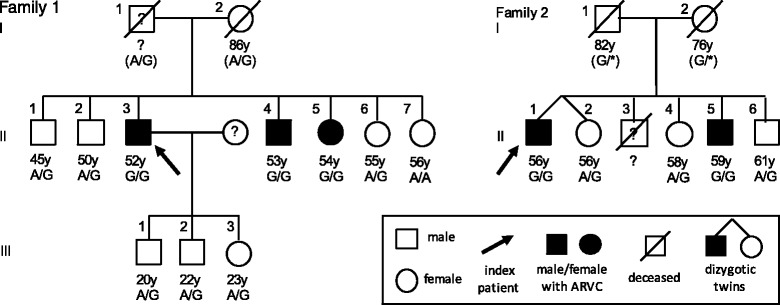
Pedigrees of the two families affected with the c.1003A > G, p.(Thr335Ala) mutation in *DSG2*. Black-filled symbols represent individuals who fulfill the 2010 Task Force Criteria by Marcus et al. [9]. Age of the family members and their genotypes are listed below the symbols. Parents of the affected individuals in family 1 are obligatory carriers of the variant. In family 2, one of them is an obligatory heterozygous carrier, while the other may either be heterozygous or homozygous for the variant

We present five homozygotes and nine heterozygotes older than 20 years from two families with this variant. The main clinical characteristics of the subjects are shown in Table 1. Pedigrees of families 1 and 2 are demonstrated in Fig. 2.

**Table 1:** Clinical characteristics of the two families with recessively inherited ARVC associated with Thr335Ala in *DSG2*

	Age (M/F)	Genotype	QRS (ms)	BBB/eps	Repolarization abnormalities	SAECG	Ventricular arrhythmias	LVEDD & EF	CMR	Dilated RV	Fulfills 2010 criteria	Clinically examined	Other
Family 1													
I.1	M	(A/G)	n.a.	n.a.	n.a.	n.a.	n.a.	n.a.	n.a.	n.a.	-	No	Deceased
I.2	86F	(A/G)	142	LBBB	No	n.a.	No	n.a.	n.a.	n.a.	No	No	Deceased Heart failure AF^a^ CAD^b^
II.1	45 M	A/G	102	No	No	n.a.	No	n.a.	n.a.	n.a.	No	Yes	
II.2	50 M	A/G	108	No	No	n.a.	No	58 mm50%	n.a.	No	No	Yes	
II.3	52 M	G/G	102	pRBBB	TI V1-V4	n.a.	Monomorphic VT of RVOT origin, VES 2800	61 mm 53%	RVEDV 322 ml RVEF 39%	Yes	Yes 2 major 1 minor	Yes	Right ventricular scar in EPS^c^ ICD^d^
II.4	53 M	G/G	92	No	TI V1-V3	LP 3/3	VES 1700	53 mm51%	RVEDVI 104 ml/m^2^ RVEF 37%	Yes	Yes 2 major 2 minor	Yes	Micro-aneurysms
II.5	54F	G/G	86	No	TI V1-V2	n.a.	Monomorphic VT of RVOT origin, VF	45 mm 52%	n.a.	Yes	Yes 1 major 2 minor	Yes	Myocardial infarction RV scar in EPS ICD
II.6	55F	A/G	88	No	No	n.a.	No	48 mm 53%	n.a.	No	No	Yes	
II.7	56F	A/A	82	No	No	n.a.	No	48 mm 70%	n.a.	No	No	Yes	
III.1	20 M	A/G	94	No	No	n.a.	No	43 mm 73%	n.a.	No	No	Yes	
III.2	22 M	A/G	88	No	No	n.a.	No	47 mm 66%	RVEDVI 76 ml/m^2^ RVEF n.a.	No	No	Yes	Collapse
III.3	23F	A/G	86	No	No	n.a.	No	46 mm 69%	RVEDVI 82 ml/m^2^ RVEF n.a.	No	No	Yes	
Family 2													
I.1	82 M	(G/^a^)	115	No	No	n.a.	VF	57 mm 40%	n.a.	No	No	No	Deceased Stroke Myocardial infarction
I.2	76F	(G/^a^)	102	pRBBB	No	n.a.	No	n.a.	n.a.	n.a.	No	No	Deceased CAD COPD^e^
II.1	56 M	G/G	108	Epsilon wave	TI II, III, aVF	LP 3/3	Monomorphic VT	54 mm 52%	RVEDVI 151 ml/m^2^ RVEF 37%	Yes	Yes 3 major 1 minor	Yes	
II.2	56F	A/G	102	No	No	n.a.	No	43 mm 78%	RVEDVI 69 ml/m^2^ RVEF 67%	No	No	Yes	
II.3	57 M	?	n.a.	n.a.	n.a.	n.a.	n.a.	n.a.	n.a.	n.a.	-	No	Deceased Pulm. Ca
II.4	58F	A/G	104	No	No	n.a.	No	48 mm 80%	n.a.	No	No	Yes	
II.5	59 M	G/G	108	No	No	LP 3/3	VES 1000, VF	53 mm 56%	RVEDVI 135 ml/m^2^ RVEF 37%	Yes	Yes 2 major 2 minor	Yes	CAD Micro-aneurysms RVOT^f^ -aneurysm
II.6	61 M	A/G	122	No	No	n.a.	No	54 mm 62%	RVEDVI 97 ml/m^2^ RVEF 64%	No	No	Yes	

Legend: Age (M/F) – age and gender (M, male; F, female); Genotype – G/G is homozygous for c.1003A > G, p.(Thr335Ala) in DSG2, A/G is heterozygous and A/A is wild type; QRS – QRS duration in milliseconds (ms); BBB/eps – presence of right/left bundle branch block (RBBB/LBBB) or epsilon wave; Repolarization abnormalities – T-inversions (TI) marked as ECG leads with abnormalities; SAECG – marked as LP for positive late potentials with the number of abnormal signal-averaged ECG 2010 Task Force Criteria fulfilled; Ventricular arrhythmias – VT for ventricular tachycardia, VF for ventricular fibrillation, number of ventricular extrasystoles (VES) per 24 h; LVEDD & EF – left ventricular end-diastolic diameter (mm) and ejection fraction (%) as measured by echocardiography; CMR – Cardiac magnetic resonance imaging data, represented by either right ventricular end-diastolic volume (RVEDV) or right ventricular end-diastolic volume index (RVEDVI) and right ventricular ejection fraction (RVEF) where applicable; Dilated RV – dilated right ventricle as measured by echocardiography or cardiac MRI by 2010 Task Force Criteria; Fulfills 2010 criteria – fulfillment of Task Force Criteria for definitive ARVC diagnosis, number or major or minor criteria fulfilled; Clinically examined – whether the person was clinically screened by the authors, or if the data provided only relies on medical records; Other – other significant clinical features

^a^Atrial fibrillation

^b^Coronary artery disease

^c^Electrophysiological study

^d^Implantable cardioverter defibrillator

^e^Chronic obstructive pulmonary disease

^f^Right ventricular outflow tract

### Family 1

Proband (II.3) is a 52-year-old male with clinical suspicion of ARVC. He had recurrent episodes of sustained ventricular tachycardia (VT) and a collapse, with the onset of symptoms at age 42. Electrophysiological study (EPS) revealed a large scar in the RV wall, and a hemodynamically unstable VT of right ventricular outflow tract (RVOT) origin was inducible. Cardiac MRI showed no abnormalities at the time of initial investigations. During the follow-up, RV enlargement was noted, and mild LV dilatation occurred without changes in systolic function or wall thickness. RV ejection fraction (EF) was diminished. Patient has an implantable cardioverter defibrillator (ICD), and is treated with beta-blockers.

Proband’s sister (II.5) is a 54-year-old female with an initial presentation of acute-onset VT with angina pain and elevated cardiac markers at age 37. Angiography reveled a coronary artery stenosis which was thought to be the culprit lesion, and percutaneous coronary intervention (PCI) was performed successfully. Thereafter, patient had recurrent events of sustained VT with no associated cardiac marker elevations and ICD was implanted. Patient later suffered an ST-elevation myocardial infarction with another PCI performed. Catheter ablation was done with no reduction in arrhythmic episodes. During the follow up, no change in LV diameter was noted, but the right ventricle underwent marked dilatation. Patient also suffered an event of ventricular fibrillation, and was resuscitated. This arrhythmia was thought to be caused by an old infarct scar. She is treated with beta-blockers and amiodarone.

Proband’s brother (II.4) is a 53-year-old male with no history of arrhythmias or other known cardiovascular disease. In the initial examination, cardiac MRI showed RV dilatation and microaneurysms in the inferior RV wall and near the RVOT, with an akinetic zone present inferiorly in the free wall. Septum was slightly hypokinetic and thickened, and in the apical septum there was signal change suggestive of myocardial fatty infiltration.

Screening of family 1 revealed altogether three homozygotes, six heterozygotes and one wild type individual (Fig. 2). All family members homozygote for the Thr335Ala fulfilled the criteria for definitive ARVC diagnosis, whereas none of the heterozygous carriers had findings relating to ARVC or any other cardiac disease. However, one of the index patient’s children (III.2) has had a collapse of unknown etiology. The wild type homozygous sibling (II.7) has had paroxysmal atrial fibrillation. The index patient’s mother, who was an obligatory heterozygous carrier of the mutation, had congestive heart failure stemming from myocardial infarction and chronic atrial fibrillation.

### Family 2

Proband (II.1) is a 56-year-old male with ARVC. He had an event of acute chest pain at the age of 51 with elevated cardiac markers. Patient was initially treated as having a non-ST elevation myocardial infarction. Subsequent angiography showed no signs of major coronary artery disease. Thereafter, patient experienced episodes of sustained monomorphic VT. Cardiac MRI showed RV dilatation and free wall akinesia. LV was also dilated and thinning of LV myocardium and hypokinesia was present. VT was inducible in EPS, but catheter ablation was not performed. Diagnostic changes were not found in endomyocardial biopsy, although immunohistochemical stains showed borderline myocarditis by Dallas criteria. Patient has an ICD, and is treated with beta blockers.

**Fig. 3:**
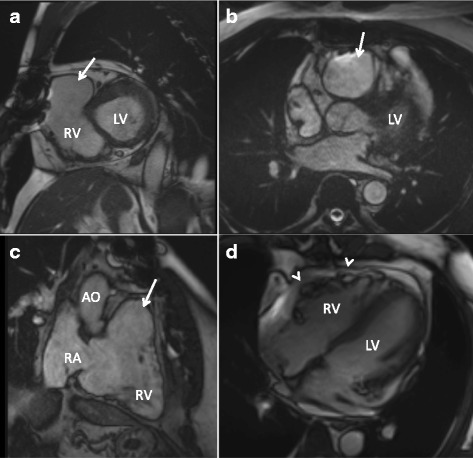
Cardiac magnetic resonance images of a 59-year-old male homozygote for the Thr335Ala in *DSG2* (II.5, family 2). Short-axis (**a**), axial (**b**) and right ventricular outflow tract (RVOT) (**c**) views of the heart show aneurysmal dilatation of the RVOT (arrows). Maximum dimension of the aneurysm was 67 mm and right ventricular end-diastolic volume index (RVEDVI) was 109 ml/m^2^. During follow-up, further dilatation of the aneurysm and the RV was observed. Four-chamber view shows rare microaneurysms in the right ventricular free wall (arrowheads) (**d**). LV = left ventricle; RV = right ventricle; RVOT = right ventricular outflow tract; RA = right atrium; AO = aorta

Proband’s brother (II.5) is 59 years old and has a history of atrial fibrillation, hypertension, hypercholesterolemia and three-vessel coronary artery disease with bypass surgery performed. Initially, cardiac MRI showed RV free wall microaneurysms, along with an RVOT aneurysm and apical dyssynchronous contraction (Fig. 3). RV was also slightly dilated and systolic function was diminished. During the follow up in a subsequent MRI, dilatation of the RV progressed markedly and the RVOT aneurysm also enlarged (diameter of 75 mm in axial plane). LV size and function was in the normal range, although it also underwent dilatation during the follow-up. Ventricular fibrillation was inducible in EPS, and ICD has been implanted.

Screening of family 2 revealed in total two homozygotes and three heterozygotes (Fig. 2). Again, both of the homozygotes fulfilled the criteria for definitive ARVC diagnosis. Heterozygote carriers had no symptoms relating to ARVC or any other cardiac disease. Neither of the index patient’s parents’ medical records showed any signs suggestive of cardiomyopathy. One of them is an obligatory heterozygous carrier of the mutation, while the other may either be heterozygous or homozygous for the variant.

## Discussion and conclusions

Based on the families described in this study and the family published by Rasmussen et al. (see below) [19], the p.(Thr335Ala) in *DSG2* associates with cardiomyopathy only in a homozygous state, as heterozygotes are considered healthy/not affected. This is consistent with recessive inheritance pattern. These findings highlight the importance of careful interpretation of the desmosomal variants. It seems that truly informative genetic counseling may require extensive clinical and genetic work-up in families with ARVC, especially when a variant is too common in control cohorts to be fully penetrant disease causing as heterozygote.

Rasmussen et al. have identified the same *DSG2* variant in a family in which two brothers had severe ARVC [19]. These brothers were homozygous for the variant whereas their healthy parents and two healthy siblings were carriers, suggestive of recessive inheritance. Detailed protein investigations demonstrated that the Thr335Ala variant protein was expressed and incorporated into desmosomes.

In the literature and open databases, the Thr335Ala in *DSG2* has been reported in ≥15 patients with ARVC (ClinVar accession SCV000060918) [25, 26, 36–44]. Family segregation is rarely studied in these reports. At least six individuals with ARVC carried an additional pathogenic *PKP2* variant [36, 37, 40–42]. We have previously detected the same *DSG2* variant as heterozygous in two patients and as homozygous in one patient. The homozygous patient was in his twenties with DCM, and did not carry any other variants considered significant for the phenotype. One heterozygous carrier was a boy who suffered sudden cardiac death, and the other was a female with clinically suspected DCM but who also carried Arg634His in *DSC2* (classified as a variant of unknown significance).

The Thr335Ala has been reported in DCM patients in two studies [20, 45]. In the first study the index patient had DCM, requiring heart transplantation at the age of 45 [20]. His brother was diagnosed with asymptomatic DCM at the age of 52. In addition to the Thr335Ala, both brothers carried p.(Glu1833Val) in *DSP* which is common in the ExAC reference population and likely has no effect on phenotype. Another brother (49y) and their mother’s sister (76y) were also carriers of these variants and they were both healthy. Two other healthy siblings (47y and 55y) carried only the Thr335Ala variant. Therefore, DCM was diagnosed in 33% of the Thr335Ala carriers in the family. Other genetic factors were recognized that probably had a role in disease penetrance. In the other study, a DCM patient had both the Thr335Ala in *DSG2* and Ala897fs*4 in *DSC2* [45]. However, the *DSC2* variant is unlikely to contribute to the phenotype, as it is also common in the ExAC database and the frameshift leads to a stop codon in the normal position.

There have also been other homozygous *DSG2* variants such as the p.(Val55Met) found in a DCM patient [46]. His parents were both carriers of the mutation but only the father had DCM. However, the father’s disease had later onset and a milder course. Immunostaining and electron microscopy of explanted LV wall myocardium from the homozygous proband revealed pale intercalated discs and significantly shorter desmosomes compared to wild-type myocardium. Unfortunately, no heterozygous carrier was studied by immunostaining. Severe disease in a patient with homozygous *DSG2* mutation and a milder disease in a carrier may indicate a dosage effect of *DSG2* mutations on cardiac function.

Variant interpretation is still challenging as earlier studies form major pitfalls by false classifications related to small reference populations, co-incidental segregations and evaluation of only a small subset of the potentially meaningful genes behind a patient’s phenotype. In diagnostic laboratories and clinics, variant classification practices are largely based on recommendations by the American College of Medical Genetics and Genomics (ACMG) [47]. Without available family segregation data, variants are classified as pathogenic only when the genotype has been identified in a certain number of patients (e.g. 5–20) with the appropriate phenotype, or when the variant has occurred de novo multiple times in association with sporadic disease. Segregation analyses of large families are considered the best approach to define causativity of a variant and assess its penetrance. Large-scale genetic research and variant sharing will eventually bring more consistency to the evaluation of families with inherited cardiac diseases.

## Additional file

Additional file 1: Descriptions of the next generation sequencing (NGS) based large panels used in the initial genetic investigations of the index patients. (PDF 21 kb)

## Abbreviations

ARVC: Arrhythmogenic right ventricular cardiomyopathy
DCM: Dilated cardiomyopathy
*DSC2*: Desmocollin-2
*DSG2*: Desmoglein-2
*DSP*: Desmoplakin
ECG: Electrocardiography
EF: Ejection fraction
EPS: Electrophysiological study
ExAC: Exome aggregation consortium
ICD: Implantable cardioverter-defibrillator
*JUP*: Plakoglobin
LV: Left ventricle
MRI: Magnetic resonance imaging
NGS: Next generation sequencing
OS-Seq: Oligonucleotide-selective sequencing
PCI: Percutaneous coronary intervention
*PKP2*: Plakophilin-2
RV: right ventricle
RVOT: Right ventricular outflow tract
SAECG: Signal-averaged electrocardiography
TFC: Task Force Criteria
VT: Ventricular tachycardia
