# Supplementary material for: Case reports of two pedigrees with recessive arrhythmogenic right ventricular cardiomyopathy associated with homozygous Thr335Ala variant in DSG2
Source: BMC Med Genet. 2017 Aug 17;18:86. doi: 10.1186/s12881-017-0442-3 (PMC5561604; doi:10.1186/s12881-017-0442-3)
Supplement: Additional file 1: — Descriptions of the next generation sequencing (NGS) based large panels used in the initial genetic investigations of the index patients. (PDF 21 kb) [file 12881_2017_442_MOESM1_ESM.pdf]

Descriptions of the next generation sequencing (NGS) based large panels used in the initial genetic investigations of the index patients.

PAN CARDIOMYOPATHY PANEL

GENES	EXONS	BASES	BASES > 15 X	MEDIAN COVERAGE	PERCENT > 15X
103	2018	408871	403041	264	98.5
GENE LIST					
ABCC9, ACADVL, ACTC1, ACTN2, AGL, ANKRD1, ATP5E, BAG3, BRAF, CALR3, CASQ2, CAV3, CBL, COA5, CRYAB, CSRP3, CTF1, CTNNA3, DES, DMD, DMPK, DNAJC19, DNM1L, DOLK, DSC2, DSG2, DSP, DTNA, EMD, EYA4, FHL1, FHL2, FKTN, FOXRED1, FXN, GAA, GATAD1, GLA, GLB1, GUSB, HFE, HRAS, ILK, JPH2, JUP, KRAS, LAMA4, LAMP2, LDB3, LMNA, MAP2K1, MAP2K2, MRPL3, MIB1, MYBPC3, MYH6, MYH7, MYL2, MYL3, MYLK2, MYOM1, MYOZ2, MYPN, NEBL, NEXN, NRAS, PDLIM3, PKP2, PLN, PRKAG2, PSEN1, PSEN2, PTPN11, RAF1, RBM20, RYR2, SCN5A, SCO2, SDHA, SGCD, SHOC2, SLC25A3, SOS1, SPRED1, SYNE1, SYNE2, TAZ, TCAP, TGFB3, TMEM43, TMEM70, TMPO, TNNC1, TNNI3, TNNT2, TPM1, TRIM63, TSFM, TTN, TTR, TXNRD2, VCL, XK					

HEART PANEL

GENES	EXONS	BASES	BASES > 15 X	MEDIAN COVERAGE	PERCENT > 15X
133	2459	489692	486847	533	99.4
GENE LIST					
ABCC9, ACADVL, ACTC1, ACTN2, AGL, AKAP9, ANK2, ANKRD1, ATP5E, BAG3, BRAF, CACNA1C, CACNA2D1, CACNB2, CALM1, CALM2, CALR3, CASQ2, CAV3, CBL, COA5, CRYAB, CSRP3, CTF1, CTNNA3, DES, DMD, DMPK, DNAJC19, DNM1L, DOLK, DPP6, DSC2, DSG2, DSP, DTNA, EMD, EYA4, FHL1, FHL2, FKTN, FOXRED1, FXN, GAA, GATAD1, GLA, GLB1, GPD1L, GUSB, HCN4, HFE, HRAS, ILK, JPH2, JUP, KCND3, KCNE1, KCNE1L, KCNE2, KCNE3, KCNH2, KCNJ2, KCNJ5, KCNJ8, KCNQ1, KRAS, LAMA4, LAMP2, LDB3, LMNA, MAP2K1, MAP2K2, MRPL3, MIB1, MYBPC3, MYH6, MYH7, MYL2, MYL3, MYLK2, MYOM1, MYOZ2, MYPN, NEBL, NEXN, NOS1AP, NRAS, PDLIM3, PKP2, PLN, PRKAG2, PSEN1, PSEN2, PTPN11, RAF1, RANGRF, RBM20, RYR2, SCN1B, SCN2B, SCN3B, SCN4B, SCN5A, SCO2, SDHA, SGCD, SHOC2, SLC25A3, SLMAP, SNTA1, SOS1, SPRED1, SYNE1, SYNE2, TAZ, TCAP, TGFB3, TMEM43, TMEM70, TMPO, TNNC1, TNNI3, TNNT2, TPM1, TRDN, TRIM63, TRPM4, TSFM, TTN, TTR, TXNRD2, VCL, XK					
